# When prevention became social: public health atomism and the assemblage of a National Immunisation Programme in the Netherlands, 1872–1959

**DOI:** 10.1017/mdh.2025.10049

**Published:** 2026-07

**Authors:** Martijn van der Meer

**Affiliations:** 1 Department of History, Erasmus School for History, Culture, and Communication, Erasmus University Rotterdam, Rotterdam, Netherlands; 2 Programme for Medical Ethics, Philosophy, and History of Medicine, Department of Public Health, Erasmus MC, Rotterdam, Netherlands

**Keywords:** Vaccination, Public Health, Chilhood Immunisation, Biopolitics, Collective Action

## Abstract

This article examines the historical transformation of childhood vaccination in the Netherlands between 1872 and 1959. It analyses how vaccination was reframed from an individual parental responsibility to a collective practice through the establishment of the ‘Rijksvaccinatieprogramma’ (National Immunisation Programme). I analyse this historical trajectory as a case of ‘public health atomism’, a strategy that achieves collective health by prioritizing individual health outcomes and local action. Rather than relying on top-down state mandates, the ‘Rijksvaccinatieprogramma’ was a consequence of co-operation between general practitioners, municipal health officials, civil society organisations, and volunteers. Drawing from published medical sources, parliamentary records, and material from local and national archives, this article provides a detailed historical account of how local governance and autonomy shaped vaccination practices, highlighting the role of the ‘entgemeenschap’ (vaccination community) as a key organisational model for situated collaboration. As such, it revisits childhood vaccination as an archetypical example of biopolitical state intervention, demonstrating how localised, flexible co-operation was instrumental in integrating vaccination into Dutch society.

## Introduction

The cover of the February issue of the *Tijdschrift voor Sociale Geneeskunde* (Journal for Social Medicine) in 1939 had never looked so sweet. It featured princess Beatrix’s face, smiling with her first two baby teeth. She had just turned one. For the editors, her birthday was not the only reason to feature her photo on the cover of their journal: ‘She had been vaccinated successfully’. The royal family had ‘given the right example’, which the editors hoped would ‘influence thousands of parents who bear the responsibility for the health of their own children’.^1^ The cover served two purposes at once. It communicated that childhood vaccination was both in the national interest and a private parental responsibility. Vaccination was at the same time individual *and* collective prevention.

This cheerful cover marked a pivotal moment. During the interwar years, most physicians and politicians perceived cowpox vaccination to help individual children avoid developing smallpox in the future as a private decision.^2^ Parents – and thus not the state – were assigned to balance fear of side effects and possible conscientious objections against the potential health benefits.^3^ Against this background, it is remarkable that by 1970, more than 90% of all parents participated in what came to be known as the *Rijksvaccinatieprogramma* (National Immunisation Programme).^4^ This scheme was coordinated by the national government and offered free routine vaccination for smallpox, polio, tetanus, whooping cough, and diphtheria. The programme has been expanded to other vaccinations, and it still exists. Even today, when vaccination rates are dropping, around 83% of all Dutch children are vaccinated through this programme.^5^ What has happened? How had childhood vaccination become so successfully interwoven in Dutch society?

In this article I argue that the transformation of Dutch childhood vaccination reflects the growing prominence of a population perspective on health, albeit in a distinctively locally anchored manner. By the Second World War, smallpox was increasingly framed not only as an individual affliction but as a threat to the nation. This aligns, at first glance, with Michel Foucault’s concept of ‘biopolitical governmentality’, wherein populations, not individuals, become the focus of state intervention.^6^ Such a perspective echoes through recent histories of childhood immunisation as the canonical case for state intervention in matters of collective health.^7^ The Dutch case, however, complicates this template. It offers an empirical account of organised childhood immunisation without presupposing a central role to play for the national government.^8^ In the Netherlands, the immunisation programme relied on co-operation between national and local governments, private physicians, public health officials, and civil society organisations. This challenges rigid analytical distinctions between ‘state’ and ‘institutions’, between ‘medicine’ and ‘public health’, but also those between the ‘individual’ and ‘population’. The contribution of this article is therefore empirical. It reconstructs how conflicting values and interests were balanced, and how an adaptive, locally brokered vaccination infrastructure carried childhood immunisation into the heart of Dutch social life.

To do so, I follow Dorothy Porter’s approach to the history of ‘public health’ as the analysis of emerging collective action aimed at achieving collective health, rather than solely as population-level health outcomes. Public health, in this sense, is about doing things together with a shared objective. From this practical perspective, it becomes essential to examine how health activities are done consistently in given space – how they, in other words, become ‘collective practices’. To subsequently analyse *how* collective practices aimed at collective health have become interwoven into the fabric of a society, I focus on the strategies that actors employed to achieve a common goal. A ‘collective strategy’ refers to the ways in which activities are coordinated to resolve context-dependent tensions – be they conceptual, political, or professional – that arise in pursuit of shared objectives. Analysing these strategies helps answer two critical questions: first, how was vaccination framed as necessary for achieving a shared objective? Second, how were vaccination efforts coordinated to align with the Dutch appreciation of the private sphere, professional autonomy, and local governance?

Crucial to my answer is a strategy I conceptualise as ‘public health atomism’. In the classic social-theory sense of *atomism versus holism*, this term refers to collective outcomes assembled from individual-level practices rather than imposed from above.^9^ I use it here historically and descriptively, not normatively,^10^ to capture how Dutch vaccination relied on the aggregation of individual decisions and locally coordinated routines ‘to lower the determinants of incidence’.^11^ That approach, as I will show, achieved population health by focusing on individual health outcomes, facilitated by locally specific co-operation between general practitioners, civil society organisations, volunteers, and municipal public health services. Rather than relying on top-down mandates, a common good was achieved through the sum of these bodily and organisational parts. By the mid-twentieth century, this atomistic approach materialised in the form of the *entgemeenschap* (vaccination community). This local organisational model facilitated co-operation between GPs and public health officials in vaccinating their communities. Moreover, these co-operations resolved key tensions by aligning national and local governance, reconciling the professional interests of GPs and public health officials, and integrating vaccination as *both* an individual and a population health intervention. In this way, public health atomism facilitated childhood vaccination into a co-operative and sustained collective practice.

In analysing this history, the paper contributes to the historiography of vaccination and offers a comparative lens for understanding public health as a collective practice.^12^ While the *Rijksvaccinatieprogramma* holds clear historiographical significance, it has received limited attention from historians of medicine. Existing accounts, mostly written by Dutch physicians and epidemiologists, focus on anniversaries or efficacy evaluations.^13^ I contribute to this literature by first tracing how childhood immunisation was shaped as a medical, political, and religious problem through an analysis of the *Nederlands tijdschrift voor Geneeskunde*, the *Tijdschrift voor Sociale Geneeskunde*, *Medisch Contact*, parliamentary documents, and published books that provide a panoramic view of the Dutch vaccination debate. Subsequently, I reconstruct the practical activities that followed from these perceptions through the archives of the *Staatstoezicht op de Volksgezondheid* (State Inspectorate of Public Health), the *Geneeskundige Hoofdinspectie* (Medical Head Inspectorate), the *Rijksinstituut voor Volksgezondheid* (Dutch Institute for Public Health), as well as relevant local city archives.

The article is structured around four parts. First, I explore how interwar fears of side effects contributed to framing vaccination as an individual responsibility.^14^ Second, I analyse how declining vaccination rates in the 1930s reframed immunisation as a collective necessity tied to military strength and national security. Third, I examine how the 1950s proliferation of *entgemeenschappen* enabled collaboration between GPs, public health officials, and civil society organisations across the Netherlands. These three parts provide building blocks for the fourth section, in which I argue that a collective strategy of public health atomism resolved political, professional, and conceptual tensions arising from the pursuit of public health through individual bodies and local collaborations.

## Part I – How vaccination became a matter of individual prevention

### The birth of indirect compulsory vaccination

Contrary to what the picture of Princess Beatrix’s may suggest, cowpox vaccination had not always been left to parental responsibility. As part of the 1872 law on infectious disease, infants were not allowed to enter public schools without a *pokkenbriefje* (a ‘pox note’). This legislation was the legal substantiation of pre-existing municipal efforts to mandate cowpox vaccination since 1814.^15^ Once cholera and smallpox epidemics hit the Netherlands in the 1860s and 1870s,^16^ the liberal government perceived the danger of infectious diseases as a ‘collective threat’ that should not be left to local authorities.^17^ According to Prime Minister Johan Rudolf Thorbecke, the vaccination requirement should protect individuals from the ‘carelessness’ of their fellow citizens in public spaces.^18^ The law became an indirect vaccination mandate with the legal introduction of compulsory schooling in 1901. Parents could no longer dodge the necessity of a pox note by keeping their children out of school.^19^ At the turn of the century, the state had unintentionally taken over what had initially been a responsibility of parents.^20^

Not everybody in the Netherlands was pleased with the indirect vaccine mandate. In response to the 1872 infectious disease bill, orthodox Protestants had already fiercely opposed the legislation in parliament and collected thousands of signatures to support a petition against ‘enforced vaccination’. Their vocal resistance offered a clear issue for broader political profiling, but other than that, these efforts were not particularly consequential.^21^ To keep attention for ‘conscientious objections’ alive, ‘anti-revolutionary’ Protestants founded the ‘League Against Enforced Vaccination’ in 1881.^22^ Their activities intensified in response to the Compulsory Schooling Act. Religious opinion makers framed vaccination as an ‘artificial intervention’ disrespecting ‘God’s Providence’. On the grounds of religious freedom, this implied that the state should enable parents to decide for themselves on vaccinating their progeny. After all – the government did not have the right to turn ‘bodies’ of its citizens into ‘objects subjected to its authority’, the League proclaimed.^23^

### A demand for absolute certainty

Liberal cabinets at the turn of the century were not particularly impressed with theological arguments. They trusted the medical science underpinning the efficacy of cowpox vaccination. However, that science could not provide the degree of certainty presumed by liberal policymakers. Johannes Pieter Schouten, a protestant physician and secretary to the League against Enforced Vaccination, was especially effective in injecting scientific doubt into the public consensus about the merits of cowpox vaccines. In various publications, he pointed out that vaccination could not account for the decline of smallpox in the nineteenth century and that statistical evidence for its efficacy was lacking. At various public lectures and newspaper interviews, Schouten insisted that hygienic measures should be more effective. He highlighted the risks of developing other diseases after vaccination, ranging from ‘measle-like irritation’, ‘fever’, and ‘general weakness’.^24^ While many physicians dismissed these claims as shape-shifting lobbying tactics,^25^ Schouten’s arguments successfully transferred the burden of proof onto the shoulders of the medical profession.^26^ Policymakers now expected science to legitimise the vaccination mandate with absolute certainty about safety.

This level of certainty proved impossible to provide. The epistemic virtue of scepticism, perceived as central to medical science at the time, clashed with the expectation of definitive proof. In 1908, Theodorus Heemskerk’s Protestant cabinet leveraged this doubt to revise vaccination policy, framing it as an opportunity to sustain ‘self-criticism’ in the medical profession. Citing Schouten, Heemskerk argued that continuing the mandate would stifle scientific inquiry and proposed allowing exemptions for children whose health risks were certified by two GPs.^27^ The resulting 1911 amendment created a loophole. It enabled parents with religious objections to bypass the pox note requirement – on *medical* grounds. In practice, this meant that if they could find a physician to state that a vaccine would be too dangerous, their child was not required to obtain one.^28^

Religious dissenters, led by Calvinist Reverend Gerrit Hendrik Kersten, capitalised on this loophole. In a 1922 publicity stunt, Kersten organised a bus trip transporting children to Calvinist GPs willing to issue medical exemptions.^29^ This act catapulted Kersten into the national spotlight and established him as a vocal opponent of vaccination mandates.^30^ By 1928, he had become a member of parliament for the S*taatkundig Gereformeerde Partij* (State Reformed Party) and successfully campaigned for the suspension of the indirect vaccination mandate. Declaring, ‘God now intervenes! Science has filed for bankruptcy’, Kersten reframed the debate as a triumph of religious freedom and individual accountability over state intervention. What had led to this dramatic pause in vaccination policy?

### A year-long pause of 12 years

While Kersten was making noise in opposing state intervention to infectious diseases, the Dutch medical profession had started to bring focus into the discussion about the side effects of cowpox vaccination – and so the meanings of ‘medical objections’ began to multiply. This began when the neurologist F. S. van Bouwdijk Bastiaanse published two case reports in January 1925 in the *Dutch Medical Journal.* He had identified a couple of patients who developed symptoms of encephalitis between 9 and 15 days after a cowpox vaccine.^31^ Van Bouwdijk Bastiaans did not intervene in an ongoing discussion in the medical literature, but it made Dutch physicians sensitive to cases of what they started to call ‘encephalitis postvaccinales’.^32^ Once they started looking for cases, they began to find them. The Dutch Medical Association initiated a working group to coordinate research in 1926,^33^ and the new Dutch Institute for Public Health quickly investigated ways to prevent or treat the side effects.^34^ Within a year, the discussion of medical objections moved from the fringe to the centre of medical discourse.^35^ Kersten followed this debate with close attention. While he initially fought the vaccination mandate to which he objected on *religious* grounds with *political* arguments about the infringement of the private sphere,^36^ the Calvinist reverend-politician quickly transported the new *medical* uncertainties to the parliamentary arena.^37^ As these medical objections gained traction, the government’s political foundation for a national vaccination policy began to shake.

Critics of vaccination used the increasing number of individual case reports on encephalitis published in medical journals to substantiate further the claim that medical science could not ‘guarantee’ that vaccines were ‘absolutely safe’.^38^ Initially, the cabinet pointed out that of all 55,000 children vaccinated in 1927, only 118 developed encephalitis: an incidence rate of one in five thousand.^39^ From a population point of view, this could have been perceived as a risk worth taking. However, Kersten prevented this epidemiologic rationale from getting accepted in parliament. He sharply criticised the prime minister’s justification, declaring that even a single case of harm was ‘already too many’. Building on this sentiment, Kersten reframed the issue as a matter of moral accountability, asserting that if one child were to die as a result of government measures, ‘the state has laid its hands on a life whose fate is determined in eternity’.^40^ For Kersten, this alone was reason enough to lift the mandate.

Other parliamentarians, social democrats, liberals, and Catholics dismissed Kerstens ominous tone of voice.^41^ Most believed that cowpox vaccination was effective and that the state was responsible for collective health. However, they agreed with Kersten that safety issues did not justify the state interfering with the private sphere of individual parents.^42^ After the Dutch Medical Association had argued that the issue of encephalitis should be investigated, the national government decided to pause the vaccination mandate as of 1928 for one year.^43^ A newly installed ‘encephalitis committee’ had to prove – preferably within a year – that cowpox vaccination was safe. This is what the committee could not deliver. They could not offer a definitive etiological explanation nor a serum that would cure any side effects. Hence, the temporary one-year pause was renewed repeatedly and would last more than 11 years.

### How vaccination became ‘individualised’

Vaccination had now returned to the domain of the household. In response, GPs saw a part to play for themselves in balancing risks and benefits for individual children, with whom they claimed to have a long-standing therapeutic relationship in their role as family physicians.^44^ The state inspector for public health agreed that vaccination ‘should be done individually’.^45^ In pulling vaccination back into the Hippocratic triangle of patient, doctor, and disease, Dutch physicians moulded the web of medical, religious, and political arguments to fit their practical realities. Vaccination should be ‘individualised’.^46^ Constitutional and developmental conditions should be carefully assessed, and medical research on cowpox vaccination should start to focus on providing information that provides input for assessment forms that would help evaluate the individual risks and help make a decision about vaccination.^47^ In the 1930s, the medical profession had agreed that ‘the vaccinating physician should adopt an individual-hygienic standpoint rather than a socio-hygienic one’.^48^ This ‘individualism’ fitted ‘the nature of the Dutch people’, an influential public health activist claimed at the Dutch Society for Social Medicine annual meeting in 1936.^49^ And so, in the first half of the twentieth century, vaccination in the Netherlands was framed less as a matter of collective prevention mandated by the national government to an issue of individual prevention undertaken by general practitioners. The focus on individualised risk assessment had moved vaccination from the domain of public health to that of medicine.^50^

## Part II – How vaccination became a matter of collective prevention

### The introduction of population immunity

The debate around vaccination again began to change when it was increasingly framed as a civic responsibility in parliamentary debates. In the first half of 1927, 99,940 bodies had been vaccinated. By the same period in 1928, the number had dropped dramatically to just 28,800.^51^ Soon, it became clear that this decline posed a threat not only to the health of individuals but also to the population. As concerns mounted, parliamentarians raised alarms over what they described as ‘the declining vaccination condition of the Dutch people’.^52^ Minister Slotemaker de Bruine acknowledged towards the end 1928 that the issue now went beyond individuals, declaring that ‘the immunity of the Dutch people as a whole’ was at risk.^53^ Declining vaccination rate became perceived as a ‘public problem:’ it had consequences for those not directly involved.^54^

A new framing of vaccination in terms of population health began to take shape in parliamentary discourse. As more cases of smallpox started to occur at the end of the 1920s, statements from the newly appointed Catholic minister for social affairs, Timotheus Verschuur, illustrate the tension between individual choice and the need for state intervention. He maintained in 1929 that ‘vaccination decisions should be made by individuals and parents with guidance from their physicians’. He requested the encephalitis advisory committee of the Dutch Health Council ‘to inform the medical community, and especially the public, about the benefits of vaccination before the age of 12 months’.^55^ The committee concluded that this would be a way to minimise the risk of encephalitis. When other physicians published research substantiating the committee’s conclusions, the state inspector for public health disseminated a pamphlet recommending that cowpox vaccination should focus on *infants.* ^56^ Physicians were expected to assess individual risks, but they also had to advocate for vaccinating infants in the name of collective health.

This reframing – from vaccination as an individual medical calculation to vaccination as a contribution to population immunity – prompted the Dutch medical profession to delineate further the conceptual relationship between individual immunity and population health more broadly. In 1935, for instance, the paediatrician who was responsible for coordinating child health care in the province of Gelderland, Isaac Alma, proposed in the *Dutch Medical Journal* to ‘call a population immune when epidemics cannot emerge’. This could be achieved if either ‘all individuals are immune’ or if the organisation of the control of infectious diseases is efficient enough to ‘[recognize] the first cases of smallpox […] and measures such as isolation, observation, and vaccination are taken’.^57^ Such a conceptual constellation urged GPs to consider not just the welfare of individual infants but also the broader interests of the population instead of merely focusing on whether a child could – or could not – be vaccinated. This resulted in a conceptual tension. If every infant were vaccinated, this would benefit ‘population immunity’ because the risk of infection would decrease, yet it may also have resulted in side effects for individuals. An individual risk calculation ‘would not be decisive for the population’, and a social risk calculation ‘would not be decisive for the individual’, as lawyer and general practitioner Schuurmans-Stekhoven pointed out in his judico-medical analysis of the ‘vaccination problem’.^58^

Public health enthusiasts working for municipal health services and child health clinics tried to account for the conflict of interest between individual and population health. They started to proclaim that ensuring population immunity might require shifting vaccination responsibilities away from GPs to public paediatricians working at child health clinics in the name of public health.^59^ This made sense, they reasoned, because the paediatricians working at these public health institutions saw almost every infant and had experience with ‘individualised’ advice on infant nutrition in the name of public health.^60^ But above all, child health clinics had recently proliferated nationwide in the 1920s. This made them an ideal venue for coordinated vaccination provision.^61^ That is, in theory. The Dutch Health Council opposed this idea on practical grounds. They argued that it would ‘overburden’ the clinics and that they would suffer from the ‘unpopularity’ of vaccines. More critically, the committee feared that transferring vaccination to health clinics would ‘antagonise the GPs’.^62^

This concern did not come out of nowhere. The conceptual tension between individual and population health that had emerged because of attention to ‘population immunity’ reflected a professional tension within the medical community. From the 1920s onwards, general practitioners increasingly viewed paediatricians working in child health clinics as competitors, fearing not only a loss of income from free consultations offered by the clinics, but also a weakening of medical prestige when contradictory advice was given in front of patients. At stake was more than a turf war: disputes over whether clinics should treat ‘sick’ infants or restrict themselves to ‘healthy’ ones, and later whether preventive child health care should be justified as serving individual or public health, cut to the heart of professional identity and jurisdiction.^63^ These unresolved tensions underscored the need for a more coordinated approach, particularly as declining vaccination rates remained a pressing public concern.

### National security at stake

GPs may have temporarily succeeded in securing the provision of vaccination as falling under their jurisdiction. Nevertheless, concerns about population immunity intensified when vaccination rates continued to drop even more in the late 1930s. Politicians began to recognise the necessity of a coordinating role for the Dutch state apparatus on a national level. This reframing of vaccination as a matter of national security and collective prevention had two reasons. First, politicians and public health officials feared that a sudden smallpox outbreak would require the organisation of mass vaccination. This would not only be a logistical nightmare. Neither careful preparation of vaccines nor the individualised assessment of risks would practically be impossible under such circumstances.^64^ Second, escalating international tensions and the looming threat of military conflict brought national defence considerations into the vaccination discourse. Minister Hendrik Slingenberg of Social Affairs warned about ‘extraordinarily challenging international political circumstances, where at any moment mass contact with other countries could occur’, noting that almost all countries around the Netherlands were ‘almost 100% immunised’. A situation that posed ‘great danger’ to the Dutch people.^65^

Critical parliamentarians were still concerned that vaccination policies would result in individual suffering. Slingenberg responded to these worries by saying that politicians in parliament ‘should not wonder what happens in the living room’. They ought to ‘focus on something entirely else, namely, what the status is of public health, of the health condition of the population as a whole’.^66^ As such, parliamentarians and public health officials reached momentum in approaching vaccination as a matter of collective prevention. Vaccination was necessary to achieve population immunity. This was in the interest of the Dutch nation. Thus, on the eve of the Second World War, the pressure mounted on the national government to end the ‘temporary pause’ on the vaccination mandate. But how?

From the perspective of the national government, the public problem of dropping vaccination rates had, compared to the nineteenth century, gained in complexity. What kind of legislation would enlarge ‘population immunity’ while respecting individual risks, conscientious objections, and the sanctity of private decision-making without being bothered by state intervention? The new Catholic minister of social affairs, Piet Aalberse, took decisive action. In January 1938, he established a state committee to advise on new vaccination legislation. To demonstrate the significance he placed on the issue, Aalberse chose to chair the committee himself. The public problem of dropping vaccination rates fitted the professional ethos of public health again.^67^ And so, Aalberse assembled an impressive roster of key figures who had shaped the institutional structure of Dutch public health in the early twentieth century. The state committee was structured to include legal and medical subcommittees which would deliberate separately and reconvene to forge a consensus on the path forward.^68^

### ‘Legally regulated coercion’ as a solution

The state committee redefined its task in terms of a question that only made sense on a population level: would it be necessary to achieve a *minimum* percentage of the population (‘minimum-immuniteitstoestand’) to get vaccinated? Answers to other questions – would this mean vaccination should be mandatory? Which individuals did not have to receive a vaccine? And was an exception still possible once the population threshold was not reached? – were perceived as dependent on this epidemiological concern.^69^ According to the medical subcommittee, a population threshold could achieve ‘population immunity’, which they explained as ‘the ratio between the number of practically completely immune individuals, those with reduced susceptibility, and those who are practically fully susceptible’. When the percentage of susceptible individuals in a group decreases, ‘the distance between the susceptible individuals increases. In the case of smallpox, the chance of spread is partly determined by this “distance”. So increasing this “distance” significantly reduces the spread of a smallpox outbreak’.^70^ In other words, the group becomes protected because enough individuals are immune, thereby reducing the chance that any remaining susceptible person encounters an infectious individual.^71^ This viewpoint had prescriptive implications: it was better to vaccinate as many individuals as possible. But *how many*?

‘That is difficult to determine’, the state committee stated without beating around the bush. The committee’s backstage minutes reveal that its members had discussed outbreaks in other countries to examine which epidemiological factors a smallpox epidemic depended on, and to calculate the vaccination index at which a population or population group remained unaffected. Yet, it turned out to be impossible to determine ‘consistently stable epidemic factors’, and ‘vaccination indexes’ often were untrustworthy.^72^ In response, the committee turned the question of the ideal vaccination degree upside down: which percentage of the population would be immune because of specific vaccination policies?

Three solutions for the public problem of dropping vaccination rates were considered. First, vaccination could remain non-compulsory, which they estimated would result in 30–40% coverage. Second, all military recruits could be revaccinated upon enlistment. Third, compulsory vaccination for all infants could be mandated, projected to yield a 50% vaccination rate after accounting for exemptions: 25% for medical reasons, 15% for conscientious objections, and 10% ‘for other reasons’.^73^ By combining infant vaccination with military revaccination, they anticipated achieving a 75% vaccination rate, the target included in the advisory report.^74^ This conceptualisation of population immunity was shaped by more than just epidemiological concerns. Military priorities and long-standing medical and religious objections deeply influenced Dutch vaccination policy, introducing inherent contradictions. Was population immunity best achieved through individual health or by managing epidemiological dynamics? Should vaccinations be the responsibility of private GPs or public health officials? And did the legislation represent top-down state intervention or a respect for individual freedoms?

The committee’s final recommendation sought to balance these tensions. It proposed a national vaccination mandate while requiring parents with objections to file for exemptions. Local mayors were tasked with meeting with objectors to discuss their concerns. Additionally, in the event of local outbreaks, the national government retained the authority to compel municipalities to implement measures. This approach aimed to encourage participation while accommodating individual and local autonomy. The committee came up with a fittingly obscure name for this balancing act. It should be referred to as *wettelijk geregelde drang* (legally regulated coercion). Practically speaking, the law created a bureaucratic hurdle for individuals using objections as an excuse. And so, on the first of January 1940, the Dutch parliament again approved a national law that mandated vaccination. Military concerns and fear of epidemics had revitalised the public dimension of vaccination coverage. Hence, after a pause of more than 11 years, vaccination had once again become a matter of collective health in which the state had a role to play. However, the new policy did not solve the political, professional, and conceptual tension that emerged as a consequence of aiming for collective prevention in a society that highly valued the local, private, and individual. The new legislation *incorporated* these tensions.

## Part III – How collective prevention was undertaken by *entgemeenschappen*

### Organised vaccination as a practical problem

Legally regulated coercion could not prevent the Dutch nation-state from capitulating to the Nazis after an armed conflict of five days – roughly four months after the new vaccination legislation came into effect. The German occupation profoundly impacted Dutch health policy. Vaccination laws were expanded to include typhoid and diphtheria,^75^ and municipal governments like The Hague facilitated large-scale immunisation campaigns.^76^ While these governments could claim reimbursement for half their expenses, the responsibility for organizing and implementing vaccinations remained largely local.^77^ After the war, when collective health deteriorated, major cities like Amsterdam, Groningen, and Rotterdam organised vaccination campaigns in collaboration with ‘cross societies’ – religiously or ideologically affiliated civil organisations with executive roles in health care. Vaccination expanded beyond smallpox to other childhood diseases, but the Dutch preference for local governance left coordination inconsistent and ad hoc. Local actors, such as child health clinics, municipal health services, and private GPs, took on central roles, but the lack of a cohesive national strategy made practical execution of organised childhood vaccination a recurring challenge.^78^

Whose responsibility was it to vaccinate? Scarce records from the years just after the war suggest three main answers. In larger cities, municipal health services offered free vaccinations through recurring sessions organised by physicians employed by the local government.^79^ In other regions, child health clinics operated by denominational ‘cross societies’ provided vaccinations for free or at a discounted rate.^80^ Third, some vaccinations were administered by GPs in their private practice. Already in 1937, the Dutch Medical Association had urged practitioners to form ‘organisations for the promotion of vaccination’ to encourage parents to bring their children in for vaccinations. Though the German occupation temporarily hindered these efforts, some of these regional organisations remained active after the occupation and continued to promote vaccinations through GPs in the late 1940s.^81^ Overall, collective action on the terrain of vaccination took place in the postwar years, but it was inconsistent, uncoordinated, and often ad hoc.

The long-standing Dutch tradition of local governance and municipal autonomy meant that coordination often remained inconsistent and ad hoc.^82^ In 1946, for instance, the municipal physician working in the municipality of Utrecht organised vaccination sessions next to a flourishing child health clinic, while general practitioners took up that role in the surrounding villages in which such clinics did not exist. They tried to convince parents during home visits and regular office hours.^83^ However, this status quo came to be perceived as problematic when outbreaks of whooping cough, diphtheria, and smallpox increasingly occurred at the end of the 1940s.^84^ Initially, national efforts to coordinate vaccinations took the shape of paper: coordination meant sending letters around. The Dutch minister of internal affairs, for example, sent a letter in March 1947 to all Dutch mayors in which he ‘requested’ to ‘take measures’ that ‘will lead to quarterly opportunities in which citizens can be vaccinated for free in your municipality’ against diphtheria. He reminded them also to take control to prevent typhoid and smallpox outbreaks.^85^ In similar vein, the state inspector for public health, Cornelis Banning, sent a letter in 1950 to all GPs to insist they revaccinate children to prevent smallpox outbreaks.^86^ These examples show how the Dutch national government initially positioned itself *alongside* local action: it may have expected local initiative, but the national government certainly did not enforce compliance. It made sure that vaccines were produced and provided free of charge, and it partially compensated the costs for needles, administration, liquids, and personnel.^87^ Nevertheless, local actors had to do the actual work: who, if at all, took on the responsibility of vaccination varied from place to place. To complicate things on the ground even further, general practitioners did often not co-operate and, at times, even *resist* these local efforts. They wanted to protect their professional domain.^88^ Vaccination thus became a boundary object – a site where competing interests clashed, and professional jurisdictions were negotiated. This fragmentation hindered the development of an effective, coordinated response to national outbreaks and undermined the possibility of achieving population immunity through collective prevention.

In search of a solution to this practical problem, the State Inspectorate for Public Health tried to bring national consistency in collective action related to vaccination in the 1950s. At the end of 1951, an advisory committee, in which representatives from the Dutch Institute for Public Health and the state inspectorate had a seat, published a ‘Report on Immunisations Against Infectious Diseases in Children’ and was sent to every Dutch physician. This ‘Blue Booklet’ – as it became known – prescribed vaccination schedules and provided a practical manual for physicians across the country. It also urged a more central role for child health clinics, as the ‘institution focused on prevention’.^89^ The governmental *Praeventiefonds* supported these recommendations by announcing a subsidy of 1 guilder per vaccination for smallpox and diphtheria, which was explicitly allocated to child health clinics.^90^ This financial backing aimed to ‘encourage the implementation of vaccinations as much as possible’.^91^

However, this sudden interference from the state reignited a familiar debate. Some GPs voiced concerns that coordination infringed on their professional autonomy.^92^ They viewed it as an intrusion into their professional domain. Public health officials, on the other side of the aisle, welcomed national coordination, which they believed was a requirement for achieving collective health.

Amidst the controversy, the southern industrial town of Tilburg emerged as a notable exception. In the early 1950s, local authorities seized the opportunity for national funding to create a cohesive vaccination strategy involving child health clinics, public health officials, and GPs. This initiative would not remain a local outlier. Rather, it became the starting point for childhood vaccination as a collective practice in the Netherlands as whole. Tilburg’s strategy exemplified how coordinated efforts at the regional level could align individual medical care with collective health goals, effectively bridging the gap between the autonomy of general practitioners and the demands of public health. It set the groundwork for a national programme that maintained the value of local governance while promoting the greater good through systematic vaccination efforts. What happened in this mid-size town in the Dutch South?

### The Tilburg model as a practical solution

It was a smallpox outbreak in 1950 forcing Tilburg’s public and private health actors to collaborate in the execution of an unprecedented mass vaccination campaign and demonstrate the potential of local partnerships for rapid public health responses. This was no easy ride. The Tilburg GPs initially only wanted to offer vaccination in their private practices at the time of the epidemics for a fee of three guilders, but given the urgency of mass vaccination in response to an ongoing epidemic, they eventually agreed to participate in a collaborative approach.^93^ Alongside nurses from cross societies, residential physicians, municipal physicians, and numerous local volunteers, GPs agreed to vaccinate in shifts between April 30 and May 3 at the office of the municipal health service at the Tilburg police station or three assigned local pubs.^94^ GPs had negotiated the right to continue vaccinating paying patients on days outside the mass vaccination campaign, and their participation was limited to a single 90-minute shift. This may seem like the bare minimum level of involvement. Nevertheless, the mass vaccination effort during the 1950 epidemic marked a turning point. During the Tilburg outbreak, physicians focused on individual patient care and those concerned with population health had experienced how effective collaboration could be.

The experiences gained from the smallpox epidemic provided a practical demonstration of how local action could meet the recommendations outlined in the Blue Booklet, transforming its abstract aspirations into actionable strategies.^95^ Tilburg physicians, drawing on this experience, had become convinced of the necessity to vaccinate against childhood diseases. In November 1952, they convened to discuss how they could organise vaccination efforts to financially compensate GPs while utilizing existing public health infrastructures. They sought to synthesise the professional domains of medicine and public health.^96^

Building on their established collaborative relations during the outbreak in 1950, GPs active in the social medicine section of the Tilburg district of the Dutch Medical Association took the initiative. They joined forces with representatives from the municipal health service, the regional inspector for public health, and leaders of Catholic and Liberal civil society organisations with whom they had learned to collaborate in the city. This unusual alliance conceived the idea to establish a ‘joint committee’ – not merely as a financial intermediary, but as a structure that would sustain co-operation in organizing and executing vaccination efforts across institutional and professional boundaries.

The bylaws of the ‘joint committee’ reveal that the actors involved *explicitly* reconciled the tension between individual and population perspectives on vaccination as a matter of collective prevention:

Based on the consideration that combating infectious diseases requires measures that serve to protect the community threatened by epidemics, and to safeguard the health interests of every individual, the GPs established in the Tilburg region of the Royal Dutch Medical Association decided to co-operate with both public and civil society health organisations, to promote the common goal of protecting the population from the occurrence of infectious diseases.^97^

A model for co-operation between individual-oriented and population-oriented vaccination professionals had been born. In practice, this strategy towards collective health meant that GPs would receive vaccines and supplies free of charge. They organised a weekly vaccination hour at child health clinics. Families of infants who had not received their vaccines by the recommended ages of 6 or 9 months would be visited by a cross-society nurse and urged to get vaccinated at the child health clinic. An administrative system was also established: based on the civil registry, parents of newborns would receive coupons for vaccines to be handed to the vaccinator – whether a GP, a nurse from a cross society, or a municipal physician. These medical professionals would then submit the coupons to the bureaucratic apparatus of the joint committee, which would file a collective claim for financial reimbursement at the national *Praeventiefonds.* ^98^ GPs were taking a risk, as the national government had not been notified in advance about central role they would play. After all, the national government had envisioned that child health clinics would do the vaccination.^99^ By February 1953, however, the state inspector for child hygiene visited Tilburg to observe the co-operative model in practice. Following his visit, the *Praeventiefonds* (National Prevention fund) approved a gross fee of 1 guilder per vaccination for all vaccinations performed under the joint committee’s coordination. This decision formalised a funding structure that reconciled local practices with national goals. It set a precedent that could facilitate proliferation across the Netherlands.^100^

At first glance, acceptance of Tilburg’s co-operative model seems a minor bureaucratic adjustment. However, considering the long history of Dutch vaccination efforts, it was a watershed moment. The Tilburg Solution reconciled three longstanding tensions: aligning individual health outcomes with collective goals, fostering joint action by GPs and public health workers, and creating a practical framework where national support empowered local action. It reframed ‘collective health’ as the sum of individual health outcomes, facilitated co-operation between GPs and public health workers, and established an effective collective strategy in which the national government supported local health infrastructures. Thus, the Tilburg solution respected and reinforced the Dutch emphasis on the individual, the private, and the local, even in the context of a common objective.

### How a local model proliferated

The public health activities in Tilburg attracted nationwide attention. In August 1953, the municipal health director of Amersfoort reached out to his counterpart in Tilburg, mentioning he had ‘heard from the state inspectorate’ about the innovative Tilburg approach.^101^ By September, the provincial child health organisation of the province of Noord-Holland was also inquiring about how Tilburg managed to involve GPs effectively.^102^ In January 1955, the ‘immunisation committee’ in The Hague drew from experiences in Tilburg and established the *entgemeenschap* (vaccination community) to experiment with a co-operative model.^103^ Unlike the Tilburg ‘joint committee’, the format in The Hague allowed GPs to vaccinate children in their own offices. Given the size of The Hague – with 250 GPs serving a population five times larger than Tilburg – the committee considered it practically impossible to organise shifts in child health clinics. GPs could also participate in the *entgemeenschap* from their private practice. Yet, the *co-operative* model that reconciled the individual and the population stood firm. And once established in The Hague, *entgemeenschappen* were transported to larger cities in the Netherlands.

By 1957, after two years of successful piloting in various local contexts, national representatives from the Dutch Medical Association, cross societies, the state inspectorate, the *Praeventiefonds*, and municipal health services met to discuss the potential of *entgemeenschappen* for the entire country for multiple reasons. They involved both private GPs and support from the *Praeventiefonds.* Unvaccinated children could be easily identified through alignment with municipal bureaucracies, and civil cross societies could conduct follow-ups by visiting the homes of unvaccinated infants. And importantly, the participants at the 1957 meeting agreed that this strategy to achieve collective prevention offered an opportunity to establish alignment across the country.^104^ Through *entgemeenschappen*, national actors wanted to ‘strive for one unified system’ that should respect local peculiarities while remaining consistent.^105^ Tilburg’s local assemblage of private practitioners, public physicians, and civil society workers provided an example for the entire country. Through the collective strategy of *entgemeenschappen*, childhood vaccination had the potential to become a collective practice.

The momentum for expanding vaccination efforts in the Netherlands grew significantly with the introduction of the polio vaccine. Although the Dutch medical community recognised polio as a severe threat, it wasn’t until Jonas Salk’s vaccine became available in the United States that the disease was viewed as an urgent public health problem in need of a solution.^106^ Initial scepticism from Dutch medical authorities about the lasting effects of an inactivated virus reinforced after the ‘Cutter incident’ in the United States – where poorly manufactured vaccines led to new polio cases – resulted in hesitations.^107^ In 1955, the Dutch Health Council advised against mass vaccination due to safety concerns,^108^ but this position changed after a national polio outbreak in 1956, which prompted politicians to worry that the Netherlands was ‘lagging behind other Western European countries’.^109^ In response, the Dutch government began importing Salk vaccines from Belgium and the United States.^110^ By May 1957, a national vaccination campaign was underway, aiming to vaccinate all children by age two – marking the first instance of state-led mass vaccination in the Netherlands.^111^ In 1959, the Dutch Institute for Public Health received government permission to produce polio vaccine itself.^112^

However, a centralised public health infrastructure that would come in handy for executing such an effort did not exist. The government decided to make polio vaccines available free of charge through the infrastructure of provincial child health organisations, which had been operational since the 1920s and supported the establishment of child health clinics.^113^ The specifics of how vaccinations were to be organised locally and how these activities would be funded were left to these provincial organisations. The government allowed flexibility but required practical consistency: vaccines could be administered by religious cross societies, municipal health services, or GPs, and it was acceptable for parents to pay for these services – although municipal funding was recommended.^114^ Thus, polio vaccination initially began as a national programme running *alongside* local efforts to vaccinate against other childhood diseases.

By 1959, the national polio vaccination campaign and local vaccination efforts through *entgemeenschappen* started to align. This was not motivated by a new political consensus about the responsibility of the national state, but by mundane bureaucratic frustrations. Physicians, nurses, and parents found it burdensome to navigate the separate administrative systems for polio and other childhood vaccines like smallpox, whooping cough, tetanus, and diphtheria. This pivoted to developing a ‘uniform administrative system’ with a ‘national vaccination booklet’ for parents, modelled after the experiences in The Hague and Tilburg, and incorporating polio vaccination.^115^ Starting in January 1959, national funding was made contingent on participation in this administrative system, prompting other regions to establish their own *entgemeenschap* with varied local alliances. Even though a co-operative model became the standard, this consistency was built on and enforced local heterogeneity.

The infrastructure of *entgemeenschappen* facilitated the transportation and standardisation of vaccination practices across the country.^116^ Once the mass vaccination campaign ended in 1960, the national government aimed to incorporate polio into ‘routine vaccination’. However, this was practically challenging due to ongoing unclarity about the ideal sequential order of different vaccines. Moreover, having multiple separate vaccines required parents to return as many as seven times for their child’s complete vaccination, demanding high discipline.^117^ To ease that burden, Dutch health authorities stuck with the injectable Salk vaccine. Besides matching the mode of alternative shots, the Salk vaccine avoided the cold-chain problems that Sabin’s alternative oral vaccine posed and could be combined with other antigens. After the Institute of Public Health received permission to make the Salk vaccine itself in 1959, childhood vaccination became even more consistent with the launch, in May 1962, of a four-component DKTP shot – diphtheria, whooping cough, tetanus, and polio. This would cut the schedule to four visits.^118^ While the DKTP vaccine was produced by the governmental Dutch Institute for Public Health and the *activity* of vaccination was funded by the Ministry for Public Health, the vaccines were injected in the arms of children by a locally varying alliance of GPs, municipal health officers, and civil society physicians.^119^ In the early 1960s, health officials and politicians started to refer to this collective action as *Het Vaccinatieprogramma van Rijkswege.* ^120^ Only later, all this work came to be remembered as what can be translated roughly as the *Rijksvaccinatieprogramma* (National Immunisation Programme).

Through a collective strategy that actors began to identify as the ‘mentality of *entgemeenschappen*’, childhood immunisation could become a collective practice.^121^ In contrast to the years immediately after the war, childhood vaccination in the early 1960s was taking place in a consistent, coordinated, and planned way. With the assemblage of the Dutch Immunisation Programme, collective prevention had become social.

## Part IV – The *entgemeenschap* as public health atomism

### Childhood vaccination as biopolitics?

The history of the *Rijksvaccinatieprogramma* provides valuable insight into the historical co-production of the state and public health.^122^ From a retrospective point of view, it is tempting to interpret Dutch childhood immunisation as the archetypical successful state intervention in population health.^123^ That interpretation mirrors Michel Foucault’s historical observations on the emergence of new modalities of power in the nineteenth century, as populations became subjects of government intervention rather than just individuals. This shift marked a transition from ‘discipline’, focusing on controlling individual behaviour, to ‘security’, aimed at managing populations through environmental regulation, statistical analysis, and risk management.^124^ In the context of vaccination, then, this transition would typically result in a shift from mandating individual vaccinations and monitoring compliance at schools or workplaces to public health campaigns, setting up vaccination centres, and using statistical models to ensure a sufficient portion of the population is vaccinated. We would thus expect that the focus on population health would lead vaccination practices to shift from what Foucault referred to as ‘institutional’ to ‘state’-led efforts.^125^

But what exactly is a population? According to Foucault, the emergence of biopower coincided with a conceptual shift. Social groups were no longer perceived as simply a ‘series’ or ‘multiplicity’ of individual bodies but as an ‘abstracted population’, made intelligible through statistical knowledge.^126^ These different perspectives on groups remain relevant in epidemiological science today, representing the distinction between researching determinants of individual cases – important for identifying high-risk individuals – and studying incidence rates – crucial for public health approaches to control the causes of disease incidence.^127^ For historians, this analytical framework has been productive for exploring ‘public health’ across different contexts – whether in various countries or different periods. Scholars like Johannes Kananen, Sophie Bergenheim, and Merle Wessel, for instance, have used the ambiguous nature of the concept of ‘public health’ – as either leaning towards the individual or the population – to investigate political and governmental shifts between institutions and states.^128^

However, epidemiologists, medical historians, and even Foucault himself have acknowledged that their broad theoretical frameworks do not always neatly align with specific historical complexities.^129^ They recognise that analytical distinctions often overlap and can be combined across different times and contexts. And indeed, the history of the Dutch National Immunisation Programme blurs distinctions between the individual and the population, medicine and public health, and between ‘institutions’ and the ‘state’. First, I have demonstrated that while biological processes on a national scale did become a concern of the national government, individual bodies remained the key focus of the doctors who performed vaccination. Moreover, and second, the national state may have supported childhood immunisation, but it did not initiate these efforts, and it continued to leave the actual execution of vaccination facilitated by civil cross organisations, GPs, and municipal physicians in organisations such as child health clinics and private practices. How, then, can we make sense of child immunisation in the Netherlands as an example of public health? Addressing this question contributes to an analytic framework to examine the historical conceptualisation of ‘population health’ as a shared objective, as well as the specific roles played by the national government and other organisations in achieving that goal across national contexts.

### Public health atomism

For the Dutch case, the answer requires to focus on how *entgemeenschappen* transformed immunisation into a collective practice. This way of doing things together, this ‘collective strategy’, helped resolve the professional and political tensions that arose from the desire for collective health in a society where local governance and respect for the private sphere were highly valued. How precisely were *entgemeenschappen* able to achieve this?

One part of the answer is that they facilitated the coordination of local collaboration. First, to actors, this co-operative model facilitated joint activity by public and private practitioners in that it offered a solution to a problem of collective action. Rather than competing for patients with their public colleagues, *entgemeenschappen* allowed GPs to contribute to a common good without making a compromise to their individual professional interests. Second, *entgemeenschappen* addressed political tensions born out of the desire to achieve collective health as a shared objective. After the Second World War, policymakers and public health officials recognised the need for national coordination to achieve collective immunity. However, the network of organisations involved in public health varied significantly depending on local contexts. In the postwar years, there was little political support for homogenizing locally diverse ways of living together.^130^ Hence, the approach of *entgemeenschappen* allowed for national coordination to achieve a shared objective, while maintaining the local heterogeneity that was required to align with Dutch society in the middle of the twentieth century.

This co-operative logic reflected an atomistic understanding of the population. Even though population health became a focus in the mid-twentieth century, its immunity was conceptualised symbiotically at both population and individual levels. By the 1960s, physicians from all backgrounds were eager to participate in *entgemeenschappen* as part of public health efforts, though their specific targets differed. While public health officials might have been content with vaccination rates of 70–80% to achieve herd immunity, GPs aimed for 100% vaccination, believing that every individual should be vaccinated, with collective health as consequence.^131^ As such, for private GPs, population immunity could remain a matter of individual prevention. In the Netherlands, the aggregate of susceptible bodies could be seen both as a ‘series of individuals’ and as an ‘abstract population’. Achieving a shared objective through the sum of organisational and bodily parts reconciled not only professional but also conceptual tensions.

This collective strategy can be conceptualised as ‘public health atomism’. By centring on individual health outcomes as the foundation for population health and leveraging pre-existing local health infrastructures to foster co-operation among general practitioners, civil society organisations, and municipal public health services, the Dutch approach exemplified an atomistic model in the sense of methodological individualism.^132^ Rather than relying on top-down mandates, public health atomism achieved the common good through the sum of its individual and organisational parts. This approach materialised in the form of *entgemeenschappen* – locally specific co-operations where diverse actors worked together to vaccinate citizens. The *entgemeenschappen* not only resolved professional, political, and conceptual tensions but also embodied the reciprocal relationship between conceptualisation and coordination: the understanding of collective health as dependent on individual participation justified a locally anchored structure, while local collaboration operationalised this framing in practice.^133^ Seen in this light, public health atomism is a historical-analytic description of how collective health was produced, not a normative endorsement of atomistic ethics.^134^ Atomism in mid-twentieth-century Dutch immunisation had the capacity to institute childhood vaccination as a co-operative, adaptable, and sustained collective practice. An analysis of *Rijksvaccinatieprogramma* as the outcome of a collective strategy thus allows for a history beyond unproductive distinctions. In the *entgemeenschap*, individual and population, medicine and public health, the national and the local all played crucial and complementary roles in making childhood immunisation across the country a practical reality.

## Conclusion

Collective health is not unequivocally the product of mandates or centralised strategies. It can as much arise from co-operation of diverse actors working towards a common goal. Examining the history of public health through the lens of collective strategies offers a valuable analytical approach – particularly when public health atomism is contrasted to what might be described as public health ‘holism’.^135^ The latter strategy, as another extreme to atomism, aims directly for collective health outcomes through large-scale, top-down, and ‘upstream’ intervention by national governments to achieve specific population metrics, such as the vaccination rates required for herd immunity. Typically, this strategy might involve mandatory vaccination laws, nationwide immunisation campaigns initiated by governments, or comprehensive public health regulations that prioritise population-level targets over individual autonomy.^136^ By contrast, atomistic strategies rely on locally anchored networks of local professionals, community initiatives, and voluntary compliance. Yet, they can still yield collective health through these vibrant patchworks of local partnerships.

Comparing collective strategies on a continuum between atomism and holism shifts focus from the *why* to the *how.* The Dutch case prompts systematic inquiry into how the ambition to achieve collective health has historically been transformed into a collective practice. As such, comparative approaches along the lines of public health atomism and holism can provide a better understanding of how various societies have sought to do health together. Rather than the structures of authority or policies handed down from above, we can follow our actors themselves in their countless practical moves, the small collaborations they set up, and the complex networks they assemble to make public health a social reality.^137^ From this viewpoint, the Dutch history of childhood vaccination demonstrates that collective health does not necessarily emerge from centralised interventions but also from vibrant patchworks of relationships and activities, facilitating co-operative public health.^138^

For example, this history also offers a complementary perspective on the relationship between medicine and public health as inherently antagonistic. As Allan Brandt and Martha Gardner have shown earlier, the twentieth century witnessed increasing tensions between these fields, with medicine becoming increasingly individualistic while public health sought to implement population-level interventions.^139^ However, the Dutch case suggests that this divide is neither inevitable nor always meaningful in practice. Instead of competition between these domains, the Dutch National Immunisation Programme evolved through co-operation, with general practitioners, public health officials, and civil society organisations working towards a shared goal. What emerged was not a clear institutional distinction between medicine and public health but an assemblage of actors co-constructing health at different scales. This resonates with Dorothy Porter’s conceptualisation of ‘social medicine’ as an evolving framework that integrates medical and societal concerns.^140^ The Dutch case invites approaching social medicine as a practical modality – one that actively facilitates co-operation between private practitioners, public health officials, and local organisations to sustain collective health.

Bringing this historical analysis into contemporary public health debates, a *practical* history of collective prevention may also challenge dominant frameworks in public health ethics. Specifically, those centring on criticism or justification of state intervention at the cost of the autonomy of individual citizens in the name of the collective good.^141^ The history of the *Rijksvaccinatieprogramma* unravels a rich tapestry of actors beyond the national government that played crucial roles in public health initiatives. Bringing those into view invites broadening ethical considerations beyond national governments: What is the value of the voluntary contributions of civil society? How to balance equity with local diversity in such co-operative arrangements? At the very least, the Dutch trajectory invites reflection on the very nature of public health itself – what it takes to act together for a common good.

## Footnotes

1

Redactie , ‘Vaccinatie Prinses Beatrix’, Tijdschrift Voor Sociale Geneeskunde [TSG], 17, 4 (1939), 25.

2 In the rest of the article, I will use the term ‘cowpox vaccination’ as this best captures the Dutch actor’s category of ‘koepokvaccinatie’. Later in the twentieth century, the notion shifted towards a more general ‘pokkenvaccinatie’ – that is why, later in the paper, I refer to ‘smallpox vaccination’.

3 ‘Handelingen Tweede Kamer 1928–1929: 27 November 1928’ (1928), 616.

4 Geneeskundige Hoofdinspectie Volksgezondheid, ‘Rapporten over de Vaccinatietoestand in Nederland, 1968–1974’, *Nationaal Archief [NA]*, 2.27.5034, 182.

5

E.A. van Lier et al., Vaccinatiegraad Rijksvaccinatieprogramma Nederland. Verslagjaar 2024 (Bilthoven: Rijksinstituut voor Volksgezondheid en Milieu (RIVM), 2024), 4.

6

M. Foucault , Security, Territory, Population: Lectures at the College De France, 1977–78 (London: Palgrave Macmillan, 2007); Claudia Huerkamp , ‘The History of Smallpox Vaccination in Germany: A First Step in the Medicalization of the General Public’, Journal of Contemporary History, 20, 4 (1985), 617–3511617294 10.1177/002200948502000407.

7 Cf. Jeffrey P. Baker , ‘Immunisation and the American Way: 4 Childhood Vaccines’, American Journal of Public Health, 90, 2 (2000), 199 10667180 10.2105/ajph.90.2.199PMC1446142; Nadja Durbach , Bodily Matters: The Anti-Vaccination Movement in England, 1853–1907 (Durham, NA: Duke University Press, 2005); James Keith Colgrove , State of Immunity: The Politics of Vaccination in Twentieth-Century America (Berkeley: University of California Press, 2006); Michael Willrich , Pox: An American History (New York: Penguin Books, 2011); Kendall Hoyt , Long Shot: Vaccines for National Defense (Cambrigde, MA: Harvard University Press, 2012); Elena Conis , Vaccine Nation: America’s Changing Relationship with Immunisation (Chicago: University of Chicago Press, 2014); Christine Holmberg , Stuart Blume , and Paul Greenough (eds), The Politics of Vaccination: A Global History (Manchester: Manchester University Press, 2017); Dóra Vargha , Polio across the Iron Curtain: Hungary’s Cold War with an Epidemic (Cambridge: Cambridge University Press, 2018)33315356; Gareth Millward , Vaccinating Britain: Mass Vaccination and the Public since the Second World War (Manchester: Manchester University Press, 2019)31487128.

8 This aligns with other counterexamples. See, for example: Peter Sköld , ‘The Key to Success: The Role of Local Government in the Organization of Smallpox Vaccination in Sweden’, Medical History, 44, 2 (2000), 201–2610829424 PMC1044252; Mary Brazelton , Mass Vaccination: Citizens’ Bodies and State Power in Modern China (Ithaca, NY: Cornell University Press, 2019); Gaëtan Thomas , ‘Keeping Vaccination Simple: Building French Immunization Schedules, 1959–1999’, Bulletin of the History of Medicine, 94, 3 (2020), 423–5833416725 10.1353/bhm.2020.0071.

9 See, for instance: J. David Lewis and Andrew J. Weigert , ‘Social Atomism, Holism, and Trust’, Sociological Quarterly, 26, 4 (1985), 455–56.

10

Joseph Heath , ‘Methodological individualism’, in E.N. Zalta and U. Nodelman (eds.), The Stanford Encyclopedia of Philosophy (Stanford, CA: Stanford University, 2024). https://plato.stanford.edu/entries/methodological-individualism/ .

11

Geoffrey Rose , ‘Sick Individuals and Sick Populations’, International Journal of Epidemiology, 14, 1 (1985), 995 10.1093/ije/14.1.323872850.

12

Dora Vargha and Imogen Wilkins , ‘Vaccination and Pandemics’, Isis, 114, 1 (2023), 50–70 .

13

D. Vos and J.H. Richardus , ‘Het Ontstaan van Het Rijksvaccinatieprogramma. Deel 1: De Vroege Jaren van Vaccinatie in Nederland, 1923–1949’, Infectieziekten Bulletin, 15, 1 (2004), 13–17 ; D. Vos and J. H. Richardus , ‘Het Ontstaan van Het Rijksvaccinatieprogramma. Deel 2: Aanloop En Totstandkoming van Het RVP, 1949–1963’, Infectieziekten Bulletin, 15, 2 (2004), 49–55 .

14 I use ‘framing’ here in the sense of Rosenberg’s and, more recently, Aronowitz’s work on the discursive process of interpreting medical and public health problems within changing cultural and political contexts. See Charles E. Rosenberg , Framing Disease: Studies in Cultural History (New Brunswick: Rutgers University Press, 1992); Robert Aronowitz , ‘Framing Disease: An Underappreciated Mechanism for the Social Patterning of Health’, Social Science & Medicine, 67 (2008), 1–9 18378372 10.1016/j.socscimed.2008.02.017.

15 For the Dutch introduction of cowpox vaccination, see: Willibrord Rutten , De Vreselijkste Aller Harpijen Pokkenepidemieën En Pokkenbestrijding in Nederland in de 18e En 19e Eeuw (‘t Goy-Houten: ES Uitgevers B.V., 1997). For international contexts, cf. Michael Bennett , War against Smallpox: Edward Jenner and the Global Spread of Vaccination (Cambridge: Cambridge University Press, 2020).

16
*Ibid.*, Rutten, 276.

17 Minister van Staat, ‘Voorziening Tegen Besmettelijke Ziekten: Memorie van Toelichting’, 1433 (1871), 19007.

18
*Ibid.*

19

N. A., ‘De wet op de vaccinatie en leerplicht’, Nederlands Tijdschrift voor Geneeskunde [NTVG], 42 (1898), 705–8; Daniëls , ‘Bond tegen vaccinedwang: consequenties leerplicht’, NTVG,44 (1900), 856 ; Daniëls , ‘Leerplichtwet contra epidemiewet’, NTVG, 45 (1901), 673 .

20 Cf: Dompeling , ‘Rapport der commissie, benoemd om na te gaan hoe de vaccinewet in verschillende gemeenten wordt toegepast, en of er behoorlijk gelegenheid tot vaccinatie wordt gegeven’, NTVG, 27 (1883), 428–33.

21

Fred Van Lieburg , ‘Revisiting the History of Religious Resistance to Vaccination in the Netherlands’, Journal for Theology and the Study of Religion, 76, 3 (2022), 225 .

22 Rutten, *op. cit.* (note 15), 276–79.

23

Rijk Kramer , Het vaccinatie-probleem (Kampen: J.H. Kok, 1913), 105 .

24

Daniëls , ‘Vaccinatiedwang: verslag lezing J.P. Schouten’, NTVG, 47 (1903), 327 ; H. Sterneberg , Vaccinatie (Hollandia-drukkerij, 1909), 24 .

25

E. Dieren , ‘Reactie op Dr. Schouten. Zal hij bij dreigend pokkengevaar zijn niet-gevaccineerde kinderen inenten, ja dan neen?’, NTVG, 47 (1903), 588–89.

26

P. Muntendam , ‘Medische bezwaren tegen vaccinatiedwang’, NTVG, 46 (1902), 1244–61; G. van Rijnberk , ‘Tegen vaccinatiedwang’, NTVG, 65 (1921), 2231 .

27 Tweede Kamer der Staten-Generaal, ‘Handelingen Tweede Kamer 1910–1911 19 Mei 1911’, 161 (1911), 2046.

28

P. Muntendam , ‘De vaccinewet’, NTVG, 55 (1911), 337 .

29 N.a., ‘Actie Tegen de Vaccinatie’, *Nieuwe Rotterdamsche Courant*, 31 March 1922; N.a. ‘Actie Tegen Vaccinatie. Een Groote Autorit’, *Het Centrum*, 31 March 1922.

30

John Exalto , Van Wie Is Het Kind? Twee Eeuwen Onderwijsvrijheid in Nederland (Amsterdam: Uitgevrij Balans, 2027), 121–24.

31 Cf. F.S. van Bouwdijk Bastiaanse , J.P. Bijl and J.Th. Terburgh , ‘Postvaccinale encephalitis’, NTVG, 70 (1926), 1267–89; A.Ch. Ruys , ‘Encephalitis in aansluiting aan vaccinatie tegen pokken’, NTVG, 69 (1925), 2807–9.

32 Cf. E. Sluiter , ‘Vaccinatie en encephalitis’, NTVG, 69 (1925), 1926–27; G. Brouwer , ‘Vaccinatie-encephalitis-influenza’, NTVG, 69 (1925), 277 ; *Ibid.*, Ruys.

33 Bouwdijk Bastiaanse *et al*., *op. cit.* (note 31).

34

F.S. van Bouwdijk Bastiaanse , ‘Vaccineserum tegen postvaccinale encephalitis’, NTVG, 70 (1926), 268–69; F.S. van Bouwdijk Bastiaanse , ‘Serumbehandeling bij encephalitis post vaccinationem’, NTVG, 70 (1926), 267–68.

35

A. Pondman , ‘Bacteriologische onderzoekingen naar aanleiding van encephalitis postvaccinalis (deel 1)’, NTVG, 71 (1927), 2037–48; A. Pondman , ‘Bacteriologische onderzoekingen naar aanleiding van encephalitis postvaccinalis (deel 2)’, NTVG, 71 (1927), 2048–55.

36 Tweede Kamer der Staten-Generaal, ‘Handelingen Tweede Kamer 1926–1927 (18 November 1926)’ (1926), 599.

37 Tweede Kamer der Staten-Generaal, ‘Aanhangsel Tweede Kamer 1926–1927 Nummer 195 VRAGEN van Den Heer Krijger Betreffende Het Optreden van Ziektegevallen Met En Zonder Doodelijken Afloop Na Inenting Met Koepokstof’ (1927); Tweede Kamer der Staten-Generaal, ‘Aanhangsel Tweede Kamer 1926–1927 Nummer 196’ (1927).

38

J.J. van Loghem , ‘Encephalitis postvaccinalis’, NTVG, 71 (1927), 872–74.

39

E. Sluiter , ‘Vaccinatie en ziektegevallen’, NTVG 71 (1927), 691 .

40 Tweede Kamer der Staten-Generaal, ‘Handelingen Tweede Kamer 1927–1928 23 December 1927, (1927), 1283.

41 Tweede Kamer der Staten-Generaal, ‘Handelingen 23 December 1927’, 1288.

42
*Ibid.*, 1291.

43 N.a., ‘Opheffing indirecte vaccinatiedwang (het antwoord van den minister)’, *NTVG*, 71 (1927), 1788; E. Sluiter , ‘Wet tot tijdelijke opheffing van den vaccinatiedwang’, NTVG, 71 (1927), 2826 .

44

I. Alma , ‘De vaccinatie tegen pokken’, NTVG, 79 (1935), 3208 .

45

J.J. van Loghem , ‘Hernieuwde opschorting van den zijdelingschen vaccinatiedwang’, NTVG, 75 (1931), 2769 .

46

Peeters , ‘Circulaire van Den Hoofdinspecteur van de Volksgezondheid Voor de Besmettelijke Ziekten No. 355/2214.26 Betreffende Vaccinatie in Het Eerst Levensjaar’, NTVG, 8, 6 (1930), 131–32.

47

M.J. van Stockum , ‘Vaccinatie tegen pokken bij zuigelingen’, NTVG, 74 (1930), 2215–24; J.J. Loghem , ‘Vaccinatie in het eerste levensjaar’, NTVG, 75 (1931), 2768–69; W. Schuurmans Stekhoven , Over pokkengevaar, vaccinatie-risico’s en inentingsdwang (The Hague: Moorman’s periodieke pers, 1937), 51–55 .

48

Nicolaas Marinus Josephus Jitta , Algemeen samenvattend rapport van de Encephalitis Commissie (The Hague: Gezondheidsraad, 1932), 278 .

49

C.J. Brenkman , ‘Gezondheidszorg Voor En Door de Bevolking: Onderling Hulpbetoon’, TSG, 14, 2 (1936), 46 .

50 Cf. Allan M. Brandt and Martha Gardner , ‘Antagonism and Accommodation: Interpreting the Relationship between Public Health and Medicine in the United States during the 20th Century’, American Journal of Public Health, 90, 5 (2000), 707 10800418 10.2105/ajph.90.5.707PMC1446218.

51 Tweede Kamer der Staten-Generaal, ‘Handelingen Tweede Kamer 1928–1929: 27 November 1928’, 611.

52
*Ibid.*, 613.

53
*Ibid.*, 617.

54 For a conceptualisation of health problems as ‘public’, see: Martijn van der Meer and Noortje Jacobs , ‘When Infant Mortality Was Born: Dutch Preventive Child Health Care without the State, 1890–1930’, European Journal for the History of Medicine and Health, 81, 2 (2024), 5.

55 Tweede Kamer der Staten-Generaal, ‘Handelingen Tweede Kamer 1929–1930 03 December 1929’ (1929), 795.

56 Peeters, ‘Circulaire van Den Hoofdinspecteur van de Volksgezondheid Voor de Besmettelijke Ziekten No. 355/2214.26 Betreffende Vaccinatie in Het Eerst Levensjaar’ (1930); H. P., ‘Vaccinatie in Het Eerste Levensjaar’, TSG, 9, 6 (1931), 106 .

57 Alma, *op. cit.* (note 44), 3206.

58 Schuurmans Stekhoven, *op. cit.* (note 47), 80.

59

R.M. van der Meer and N. Jacobs , ‘In the name of the public: How the medical marketplace helped to redefine Dutch preventive child health care, 1920–1940’, in Physicians and Society in a Transforming Europe (1750–1950) (Milton Park: Routledge, forthcoming).

60

J.J. van Loghem , ‘Nieuw denkbeeld ter voorkoming van encephalitis postvaccinalis’, NTVG, 78 (1934), 5401 ; J.J. van Loghem , ‘De besmettelijke ziekten in het jaar 1932’, Nederlands Tijdschrift voor de Geneeskunde, 78 (1934), 3608–10.

61 R.M. van der Meer, F.R.R. van der Zee, and Noortje Jacobs, ‘A Good Mother Enters the Clinic: How a Preventive Institution became an Agent of Socialization’, *Bulletin for the History of Medicine* (in press).

62

J.J. van Loghem and R. P. van de Kasteele , ‘Rapport Commissie Prophylactische Inentingen’, TSG,14, 2 (1936), 41 .

63 Van der Meer and Jacobs, *op. cit.* (note 59).

64

Tweede Kamer der Staten-Generaal, ‘Verlenging van Den Geldigheidsduur van Tijdelijke Bepalingen Betreffende de Vaccinatie, Enz.; Voorlopig verslag’ (1936), 254 .

65 Eerste Kamer der Staten Generaal, ‘Handelingen Eerste Kamer 1936–1937; 30 December 1936’ (1936), 93.

66 Citation of Slingenberg in a debate in the Dutch senate. See Schuurmans Stekhoven, *op. cit.* (note 47), 79.

67 Brandt and Gardner, *op. cit.* (note 50), 707.

68

Cath.M. Schenck , ‘Het vaccinatievraagstuk (instelling staatscommissie)’, NTVG, 82 (1938), 1038 .

69

C.P.M. Romme , Verslag van de staatscommissie inzake de vaccinatie (The Hague, Algemeene Landsdrukkerij, 1939), 9 .

70
*Ibid.*, 114.

71 Cf. David S. Jones , ‘Mischievous Immunity: How Intellectual History Can Be Relevant for Current Public Health Practice’, American Journal of Public Health, 111, 8 (2021), 1376–78, 634464205 10.2105/AJPH.2021.306356PMC8489623; David Robertson , ‘Of Mice and Schoolchildren: A Conceptual History of Herd Immunity’, American Journal of Public Health, 111, 8 (2021), 1473–8034111938 10.2105/AJPH.2021.306264PMC8489650; Warwick Anderson , ‘Immunities of the Herd in Peace, War, and COVID-19’, American Journal of Public Health, 112, 10 (2022), 1465–7035926163 10.2105/AJPH.2022.306931PMC9480480.

72 Romme, *op. cit.* (note 69), 116.

73
*Ibid.*, 125.

74
*Ibid.*, 129.

75 Geneeskundige hoofdinspectie, ‘Staatsblad’, pagina 250. Stukken Betreffende de Bestrijding van Besmettelijke Ziekten En de Organisatie van Die Bestrijding’, *NA*, 2.15.38, 163.

76

L. W. Rümke and H. C. Rümke , ‘Difterie: Een ‘Vergeten’ Ziekte?’, NTVG, 163 (2019), 2.31609562.

77

Vereniging Nederlandsche Gemeenten, ‘Officiële Bekendmaakingen IX:40, No 11450’, Regionaal Archief Tilburg [RAT], 81.2.3,7, 83 (1943).

78 See, for example, J.J. Brutel de la Rivière and F. Wibaut , ‘Verzoekschrift Aan de Kabinetsformateur’, Medisch Contact, 8, 1 (1946), 185–87; As quoted in: H.P. Verbrugge , J.A. van Vliet , and H. Rümke , ‘Hoe Begon Het Rijksvaccinatieprogramma Voor Kinderen? Lessen Voor de Vaccinatie van Volwassenen’, Infectieziekten Bulletin, 1 (2025). https://www.rivm.nl/weblog/IB-hoe-begon-rijksvaccinatieprogramma-voor-kinderen-lessen-voor-vaccinatie-van-volwassenen-IB0125 .

79

R.E. Wieringa , ‘De Resultaten van 7 Jaren Toepassing van de Inentingswet 1939 in de Stad Utrecht’, TSG, 18 (1947), 290–91; Geneeskundige hoofdinspectie, ‘Stukken Betreffende Een Handleiding Voor Geneeskundigen Inzake Maatregelen Bij Besmettelijke Ziekten’, *NA*, 2.15.38, 168.

80

D. Hemmes , ‘Vaccinatie Tegen Pokken in de Provincies Utrecht En Overijssel Vóór En Na de Invoering van de Inentingswet 1939’, TSG, 18 (1947), 288–90.

81

E. Sluiter , ‘Vaccinatie tegen pokken na de toepassing der inentingswet in de stad Utrecht en de provincies Utrecht en Overijssel’, NTVG, 91 (1947), 3164 .

82 Cf. Staf Hellemans , ‘Pillarization (‘Verzuiling’). On Organized “Self-Contained Worlds” in the Modern World’, The American Sociologist, 51 (2020), 1–24 ; A. Lijphart , The Politics of Accommodation: Pluralism and Democracy in the Netherlands (Oakland: University of California Press, 1968).

83 Hemmes, *op. cit.* (note 80), 288.

84 See, for example, J.J. Brutel de la Rivière and F. Wibaut , ‘Verzoekschrift Aan de Kabinetsformateur’, Medisch Contact, 8, 1 (1946), 185–87. As quoted in H.P. Verbrugge , J.A. van Vliet and H. Rümke , ‘Hoe Begon Het Rijksvaccinatieprogramma Voor Kinderen? Lessen Voor de Vaccinatie van Volwassenen’, Infectieziekten Bulletin, 1 (2025), https://www.rivm.nl/weblog/IB-hoe-begon-rijksvaccinatieprogramma-voor-kinderen-lessen-voor-vaccinatie-van-volwassenen-IB0125.

85 Geneeskundige hoofdinspectie, ‘Stukken Betreffende Een Handleiding Voor Geneeskundigen Inzake Maatregelen Bij Besmettelijke Ziekten’ Letter, dd. 27 March 1947.

86

E. van der Werf , ‘De duur van de vaccinatie-immuniteit bij pokken’, NTVG, 94 (1950), 383–85.

87 De Minister van Sociale Zaken, ‘Letter, titled “Toekening Rjksbijdrage gemeente Tilburg,” (dd 18 July 1949)’ in RAT 81.2.3,7, 83 (1943).

88

G.C. Heringa and W. A. Weisfelt , ‘Over koepokinenting en nog wat’, Medisch Contact, 49 (1948), 971 .

89 With help of Hans Rümke, I have only been able to locate a few pages of the original report. For the quotes, I rely on two contemporary publications: I.A. van Goor , ‘Huisarts En Preventieve Gezondheidszorg’ (Leiden University, 1961); and J.J. van Loghem , ‘De taak der consultatiebureau’s voor zuigelingen in de strijd tegen de besmettelijke ziekten’, NTVG, 95 (1951), 3453 .

90
*Ibid.*, Goor, 73.

91

G.C. Heringa , ‘Het Immunisatie Rapport En de Taak Der Huisartsen’, Medisch Contact, 6 (1951), 616–19; Loghem, ‘De taak der consultatiebureau’s voor zuigelingen in de strijd tegen de besmettelijke ziekten’; P.J.M. Haanappel , ‘Consultatiebureaux En Inentingen’, TSG, 25 (1951), 471–73.

92

G.C. Heringa , ‘Het Immunisatie rapport en de taak der huisartsen’, MC, 6 1951 (616–9).

93 A. Sluyters, ‘Bericht aan het Dagelijks Bestuur, naar aanleiding van zijn bezoek aan Tilburg, in verband met berichten minder juiste handelingen bij vaccinatie (dd. 16 May 1951)’, in: RAT 81.2.3.6, 181.

94 S. Ausems, ‘Letter to Local Physicians on Vaccination Organization during the 1951 Smallpox Outbreak (dd. 3 May 1951)’, in: RAT 81.2.3.6, 180.

95 Commissie van Inspecteurs der Volksgezondheid, hoogleraren en Specialisten in de Kindergeneeskunde, ‘Organisatie der Uitvoering in Tilburg en Omgeving van het Rapport: Immunisatie tegen infectie ziekten bij kinderen (dd. 1951), page 1’, in RAT: 81.2.3.7, 81.

96
*Ibid.*

97 Commissie van Overleg inzake de bestrijding van besmettelijke ziekten in Tilburg, ‘statuten en regelement’, in: RAT 81.2.3.7, 81.

98 Commissie van Inspecteurs der Volksgezondheid, hoogleraren en Specialisten in de Kindergeneeskunde, ‘Organisatie der Uitvoering in Tilburg en Omgeving van het Rapport: Immunisatie tegen infectie ziekten bij kinderen (dd. 1951), pages 2–4’, in: RAT 81.2.3.7, 81.

99 J.A.A. van de Horst ‘Beschrijving van de commissie van overleg inzake de vestrijding van besmettelijke ziekten’, in RAT 81.2.3.7, 81.

100 I.A. van Goor, ‘Letter on the Financial Aspects of Immunisation, Addressed to the Sectie Sociale Geneeskune, Kring Tilburg en Omstreken (dd. 17 March 1953)’, in: RAT 81.2.3.7, 81.

101 Directeur van de GG&GD te Amersfoort, ‘Letter Addressed to J.A.A. van der Horst (dd. 11 August 1953)’, in: RAT 81.2.3.7, 81.

102 Secretaresse der Stichting voor Moederschapszorg en Kinderhygiëne in Noordholland, ‘Letter Addressed to o J.A.A. van der Horst (dd. 12 September 1953)’, in: RAT 81.2.3.7, 81.

103 Geneeskundig Inspecteur van de Volksgezondheid, ‘Letter titled “Immunisatie” (dd. 13 October 1951)’, in: RAT 81.2.3.7, 81; J.P. van der Schroeff, ‘Entgemeenschap ‘s Gravenhage’, in: RAT 81.2.3.7, 81.

104 Geneeskundige Hoofdinspectie, ‘Kort verslag van de bespreking betreffende “Enggemeenschappen”, gehouden op de Geneeskundige Hoofdinspectie van de Volksgezondheids te ‘s-Gravenhage, op woensdag 23 Januari 1957 om 14.00 (page 1)’, in: RAT 81.2.3.7, 81.

105 Geneeskundige Hoofdinspectie, ‘Kort verslag van de bespreking betreffende “Enggemeenschappen” (page 6)’, in: RAT 81.2.3.7, 81.

106 Cf. David Oshinsky , Polio: An American Story (Oxford: Oxford University Press, 2005).

107

M. F. Polak , ‘De werkzaamheid van een geïnactiveerd poliomyelitisvirus als vaccine’, Nederlands Tijdschrift voor Geneeskunde, 99 (April 1955), 1847–50; Stuart S. Blume , ‘Lock in, the State and Vaccine Development: Lessons from the History of the Polio Vaccines’, Research Policy, 34, 2 (2005), 163 .

108 Ch. A. Hannik , Over de Vaccinatiecampagne Tegen Poliomyelitis Gedurende de Jaren 1957–1962 (’s-Gravenhage: Staatsdrukkerij- en uitgeverijbedrijf, 1963), 5–7 ; N. A., ‘Inenting Tegen Kinderverlamming’, TSG, 12 (1956), 350 13355839.

109

Tweede Kamer der Staten-Generaal, ‘Wijziging van Hoofdstuk XII Der Rijksbegroting Voor Het Dienstjaar 1956; voorlopig verslag’, 413 (1956), 1.

110 Blume, *op. cit.* (note 107), 167; Redactie , ‘Salk-vaccine naar Nederland?’, Nederlands Tijdschrift voor Geneeskunde, 101 (1956), 95 .

111

M. M. Hilfman , ‘Persconferentie over de voorgenomen vaccinatie tegen poliomyelitis’, NTVG, 101 (1957), 988 ; Hannik, *op. cit.* (note 108), 17.

112 Blume, *op. cit.* (note 107), 167.

113

C. Banning , ‘Organisatie van landelijke immunisatie tegen poliomyelitis’, NTVG, 101 (1957), 94–95 .

114
*Ibid.*, 96.

115 Chr.J.M. Mol and H.P. Matthee, ‘Provinciale Raad voor Gezondheidszorg in Noord-Brabant (dd. 10 November 1958)’, in RAT 81.2.3.7, 81; W. A. van Goor , ‘Het Nieuwe Inentingsboekje’, Medisch Contact, 7 (1959), 90–92 .

116 Thomas, *op. cit.* (note 8).

117 Directoraat-Generaal Volksgezondheid van het Ministerie van Welzijn, Volksgezondheid en Cultuur, ‘“Nota over Het Polio-Vaccinatie-Beleid”, Opgesteld Door de Geneeskundige Hoofdinspecteur van de Volksgezondheid,’ in NA 2.15.65, 1437.

118

C. F. Brenkman , ‘Toepassing van het vierfactoren-vaccin: een veelbelovend project’, Medisch Contact, 17 (1962), 270 ; Blume, *op. cit.* (note 107).

119 Directeur van de Volksgezondheid, ‘memo to the minister, titled “Vaccinatie Poliomyelitis” (dd. 18 April 1957)’, in NA 2.27.5034, 181.

120 H.J. Dijkhuis, ‘Nota van de Geneeskundig Hoofdinspecteur over de organisatie en financiering bij toepassing van het vier-factorenvaccin (dd. November 1961), page 3’, in NA 2.27.5034, 181.

121 This quote is a translation for ‘entgemeenschap-gedachte’. See Brenkman, *op. cit.* (note 118), 268.

122

Dorothy Porter , The History of Public Health and the Modern State (Amsterdam: Rodopi, 1994)7526977; Dorothy Porter , Health, Civilization and the State: A History of Public Health from Ancient to Modern Times (Milton Park: Routledge, 2005)10.1136/bmj.318.7190.1083PMC111547710205130.

123 This is how the programme is remembered in the Netherlands. See Vos and Richardus, *op. cit.* (note 13), ‘Het Ontstaan van Het Rijksvaccinatieprogramma. Deel 2: Aanloop En Totstandkoming van Het RVP, 1949–1963’; L. Kamp , H.E. de Melker and J. A. van Vliet , ‘Rijksvaccinatieprogramma: is minder vaccineren een optie?’, NTVG, 164 (2020)33030320; T. M. Schurink-van T Klooster et al., ‘De ontwikkeling van het Rijksvaccinatieprogramma in Nederland’, JGZ Tijdschrift voor jeugdgezondheidszorg, 51, 3 (2019), 54–60 .

124

Michel Foucault , Mauro Bertani and François Ewald (transl.), Society Must Be Defended : Lectures at the Collège de France, 1975–76 (London: Penguin, 2004), 249–50.

125
*Ibid.*

126 Foucault, *op. cit.* (note 6), 12.

127

Cf. Geoffrey Rose , ‘Sick Individuals and Sick Populations’, International Journal of Epidemiology, 30, 3 (2001), 427–32711416056 10.1093/ije/30.3.427.

128

Johannes Kananen , Sophy Bergenheim and Merle Wessel , Conceptualising Public Health: Historical and Contemporary Struggles over Key Concepts (Milton Park: Routledge, 2018), 1–17 .

129 Foucault, *op. cit.* (note 6), 12; Rose, *op. cit.* (note 127), 432.

130 Cf. Staf Hellemans , ‘Pillarization (‘Verzuiling’). On Organized ‘Self-Contained Worlds’ in the Modern World’, The American Sociologist, 51 (2020), 1–24 .

131 Cf. G. Dekker , ‘Inentingen tegen polio-myelitis’, Medisch Contact, 21 (1957), 358–59; G. Dekker , ‘Van het hoofdbestuur: De Polio-vaccinatie en de huisarts’, Medisch Contact, 20 (1957), 341–42.

132

Lewis, J. David , ‘Social Atomism, Holism, and Trust’, Sociological Theory, 3, 1 (1985), 1–12 ; Heath, *op. cit.* (note 10).

133 The lens of public health atomism enables comparing the *entgemeenschappen* with similar organizational structures elsewhere. In New York City, for instance, voluntary district-level partnerships between paediatricians and civic groups proved crucial in diphtheria immunization campaigns in the 1920s. See Colgrove, *op. cit.* (note 7), ‘Chapter 3 – Diphtheria Immunization: the Power, and the Limits, of Persuasion’. Another example may be Sweden’s vaccination districts likewise enabled a physician, a midwife, or a parish clerk to administer vaccines. See J. Rogers Hollingsworth et al., State Intervention in Medical Care: Consequences for Britain, France, Sweden, and the United States, 1890–1970 (Ithaca, NY: Cornell University Press, 2019), 79–82 . Set beside these cases, Dutch *entgemeenschappen* stand out for the way they institutionalised local self-rule within a nationwide funding frame.

134 See, for a classic example of critiquing atomistic ethics: Michael J. Sandel , Liberalism and the Limits of Justice (Cambridge: Cambridge University Press, 1982).

135 Cf. Rick J. Carlson , ‘Holism and Reductionism as Perspectives in Medicine and Patient Care’, Western Journal of Medicine, 131, 6 (1979), 466 397674 PMC1271903; Vijay Kumar Yadavendu , Shifting Paradigms in Public Health: From Holism to Individualism (New Delhi: Springer India, 2013); Valerie Michaelson , William Pickett , and Colleen Davison , ‘The History and Promise of Holism in Health Promotion’, Health Promotion International, 34, 4 (2019), 824–3229897526 10.1093/heapro/day039.

136 See, for example: David Arnold , Colonizing the Body: State Medicine and Epidemic Disease in Nineteenth-Century India (Oakland, CA: University of California Press, 1993); Dóra Vargha , Polio Across the Iron Curtain: Hungary’s Cold War with an Epidemic, Global Health Histories (Cambridge: Cambridge University Press, 2018)33315356.

137

I have borrowed this phrasing from Bruno Latour , Reassembling the Social: An Introduction to Actor-Network-Theory (Oxford: Oxford University Press, 2007), 12 .

138 This echoes the anarchist histories of public health. Cf., Richard Morgan , The Making of Kropotkin’s Anarchist Thought: Disease, Degeneration, Health and the Bio-Political Dimension (Milton Park: Routledge, 2020); Tony Parker and Jane E Ferrie , ‘Health and Welfare: Rejecting the State in the Status Quo – Examples of an Anarchist Approach’, International Journal of Epidemiology, 45, 6 (2016), 1754–5828538989 10.1093/ije/dyx001; Niall Scott , ‘Anarchism and Health’, Cambridge Quarterly of Healthcare Ethics, 27, 2 (2018), 217–2729509120 10.1017/S0963180117000561.

139 Brandt and Gardner, *op. cit.* (note 50).

140

Dorothy Porter , ‘How Did Social Medicine Evolve, and Where Is It Heading?’, PLoS Medicine, 3, 10 (2006), e399. 10.1371/journal.pmed.0030399 17076552 PMC1621092.

141 Cf. Thomas Nys , ‘Paternalism in Public Health Care’, Public Health Ethics, 1, 1 (2008), 64–72 ; Ruth Faden , Justin Bernstein , and Sirine Shebaya , ‘Public Health Ethics’, in Edward N. Zalta (ed.), The Stanford Encyclopedia of Philosophy (Stanford, CA: Stanford University, 2022); Angus Dawson and Marcel Verweij (eds.), Ethics, Prevention, and Public Health (Oxford ; New York: Clarendon Press, 2009); Roland Pierik and Marcel Verweij , Inducing Immunity?: Justifying Immunisation Policies in Times of Vaccine Hesitancy (Cambridge, MA: MIT Press, 2024)10.1093/phe/phag016PMC1335694042438464.

